# Seascape genomics reveals population isolation in the reef-building honeycomb worm, *Sabellaria alveolata* (L.)

**DOI:** 10.1186/s12862-020-01658-9

**Published:** 2020-08-10

**Authors:** Anna P. Muir, Stanislas F. Dubois, Rebecca E. Ross, Louise B. Firth, Antony M. Knights, Fernando P. Lima, Rui Seabra, Erwan Corre, Gildas Le Corguillé, Flavia L. D. Nunes

**Affiliations:** 1 Conservation Biology Research Group, Department of Biological Sciences, University of Chester, Parkgate Road, Chester, CH1 4BJ UK; 2 Laboratoire des Sciences de l’Environnement Marin, LEMAR UMR 6539 CNRS/UBO/IRD/Ifremer, Université de Brest (UBO), Université Européenne de Bretagne (UEB), Institut Universitaire Européen de la Mer (IUEM), 29280 Plouzané, France; 3 Ifremer, DYNECO, Laboratory of Coastal Benthic Ecology, F-29280 Plouzané, France; 4 Marine Biology and Ecology Research Centre, School of Biological and Marine Sciences, University of Plymouth, Plymouth, PL4 8AA UK; 5 Institute of Marine Research, 1870 Nordnes, 5817 Bergen, Norway; 6 School of Biological and Marine Sciences, University of Plymouth, Plymouth, PL4 8AA UK; 7 CIBIO/InBIO, Centro de Investigação em Biodiversidade e Recursos Genéticos, Universidade do Porto, Vairão, Portugal; 8 CNRS, Sorbonne Université, FR2424, ABiMS, Station Biologique de Roscoff, 29680 Roscoff, France

**Keywords:** RADseq, Ocean circulation modelling, Adaptation, Marine invertebrate, Larval dispersal

## Abstract

**Background:**

Under the threat of climate change populations can disperse, acclimatise or evolve in order to avoid fitness loss. In light of this, it is important to understand neutral gene flow patterns as a measure of dispersal potential, but also adaptive genetic variation as a measure of evolutionary potential. In order to assess genetic variation and how this relates to environment in the honeycomb worm (*Sabellaria alveolata* (L.)), a reef-building polychaete that supports high biodiversity, we carried out RAD sequencing using individuals from along its complete latitudinal range. Patterns of neutral population genetic structure were compared to larval dispersal as predicted by ocean circulation modelling, and outlier analyses and genotype-environment association tests were used to attempt to identify loci under selection in relation to local temperature data.

**Results:**

We genotyped 482 filtered SNPs, from 68 individuals across nine sites, 27 of which were identified as outliers using BAYESCAN and ARLEQUIN. All outlier loci were potentially under balancing selection, despite previous evidence of local adaptation in the system. Limited gene flow was observed among reef-sites (F_ST_ = 0.28 ± 0.10), in line with the low dispersal potential identified by the larval dispersal models. The North Atlantic reef emerged as a distinct population and this was linked to high local larval retention and the effect of the North Atlantic Current on dispersal.

**Conclusions:**

As an isolated population, with limited potential for natural genetic or demographic augmentation from other reefs, the North Atlantic site warrants conservation attention in order to preserve not only this species, but above all the crucial functional ecological roles that are associated with their bioconstructions. Our study highlights the utility of using seascape genomics to identify populations of conservation concern.

## Background

Conservation strategies in the context of climate change rest on the premise that populations can show three responses to avoid fitness loss: evade, evolve or acclimatise [1]. Characterisation of genetic diversity can shed light on these responses [2], by indicating dispersal and gene flow using neutral genetic markers (evasion), or adaptation using adaptive genetic variation, which inform us about the potential for an organism to evolve in altered conditions [3–5]. Separating neutral and adaptive causes of phenotypic differentiation, or lack thereof in a heterogeneous environment (sensu countergradient variation [6];), can require logistically difficult experiments [7]. However, advances in genomic technologies and analyses have opened up new avenues for exploring genome-wide (neutral) versus locus-specific (adaptive) genetic variation between populations, allowing us to compare patterns of adaptive genetic diversity with environmental variation to infer agents of selection [8, 9]. These methods have already been successful in contrasting neutral and non-neutral genetic variation in a range of taxa including fish [5, 8, 10, 11], insects [4], and marine invertebrates [12, 13] and represent an important step in interpreting genetic diversity in relation to environment.

Despite genomic advances allowing investigations of genetic diversity in non-model species, which is particularly important for species of conservation concern, understanding of gene flow and local adaptation in marine environments had, until recently, lagged behind that for terrestrial species [14–16]. Recent advances in seascape genomics have facilitated consideration of the effect of landscape on neutral and adaptive genetic variation in marine environments, taking into account ocean circulation patterns and environmental variation to explain observed population structuring [8, 17]. For instance, Benestan et al. (2016) [17] found that ocean currents as well as geographic distance were key to explaining observed patterns of neutral genetic variation in the American lobster (*Homarus americanus* H. Milne Edwards, 1837), whereas sea surface temperature acted as an agent for natural selection. Ocean circulation modelling for understanding dispersing larval stages has emerged as an important tool to explain neutral population structure where isolation-by-distance measures alone are uninformative [18–20].

Intertidal communities have been proposed as particularly suitable for studying the impacts of climate change, as many species already exist at the limit of their thermal tolerance ranges [21, 22]. Ecosystem engineers provide and modify habitat for other species and are thus disproportionately important to diversity, ecological functioning, and conservation in a changing climate [1, 14, 23–25]. Therefore, understanding how littoral ecosystem engineers have adapted to the local environment they experience is key to understanding not only their future survival in a changing climate, but also the potential survival of intertidal ecosystems as a whole.

The honeycomb worm (*Sabellaria alveolata* (Linneaus, 1767)), is an ecosystem engineer that constructs biogenic reefs along temperate coastlines of North Africa and Europe [26]. They are gonochoric sexually reproducing broadcast spawners. Asexual reproduction is not known to occur in the species, in line with the observation that clonality occurs in less than 1% of polychaetes [27]. Larvae are dispersive but preferentially settle on pre-existing reef or remains, although they do not discriminate based on reef or origin [28, 29]. Each individual constructs a tube from sand, which is glued together with biomineralised cement [29, 30]. Individuals are gregarious and tubes aggregate to form large bioconstructions or reefs, which can stretch over several kilometres [31–33]. These reefs provide a large diversity of microhabitats for other species and alter wave action, water circulation, erosion and sedimentation patterns [34], and phytoplankton concentration [35] and thus modify the availability of resources, leading to a high associated biodiversity compared to surrounding areas [36, 37]. Biogenic reefs, including those of *Sabellaria*, are listed under the EC Habitats Directive (92/43/EEC) Annex 1 and are thus of conservation interest [38]. Despite this, little is known about neutral genetic structure (but see [39, 40]) and to our knowledge nothing is known about adaptive genetic structure of *S. alveolata*. Neutral population structuring is expected because (i) adults are sessile, and (ii) during the dispersive planktotrophic larval stage of this species, individuals have been shown to demonstrate positive taxis towards the cement of sand grains and bioclasts of *S. alveolata* tubes [41] leading to site philopatry [42]. However, modelling of larval dispersal is needed to fully understanding how the environment influences gene flow within marine systems [17].

We have previously demonstrated that *S. alveolata* show local adaptation to temperature in terms of their membrane lipid composition [43] but the genomic basis of this adaptation is not known. Therefore, as their distributional range extends along a latitudinal thermal gradient from Scotland to Morocco, *S. alveolata* provide an excellent opportunity to investigate if/how adaptive population structure is related to temperature, and assess the role of neutral processes in gene flow.

In this study, we explore the extent of neutral and adaptive genetic variation among reef sites of the honeycomb worm, *S. alveolata*, and the relationship with the environment. We test: 1) if gene flow is restricted among *S. alveolata* reef sites and whether population structure can be predicted by ocean circulation modelling; and 2) whether *S. alveolata* show evidence of local adaptation and if this relates to local thermal conditions.

## Results

### SNP detection and summary statistics

**Fig. 1:**
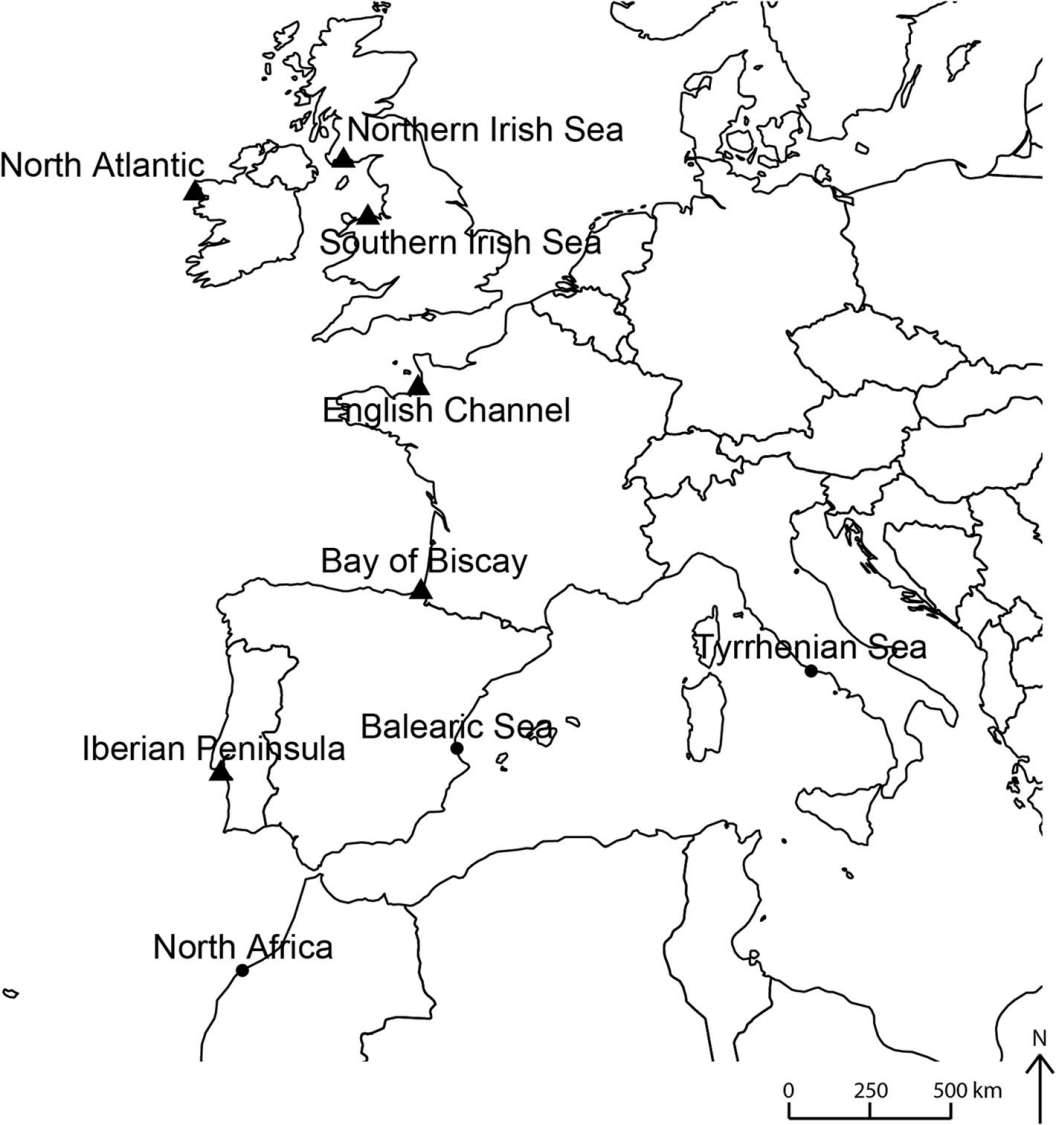
Sampling sites spanning the latitudinal and longitudinal range of *S. alveolata*, which were also used as the release sites for the Lagangrian model*.* Sites shown as triangles are those from which temperature was available. Created in R version 3.1.2 [44]

Due to high variability in sequence coverage between samples (37,000 - 1,400,000 reads per sample; Additional file 1), only 68 samples across the nine sites were retained for analysis (Table 1). Of these, 50 samples were from sites where temperature data was also available (Table 1; Fig. 1). In total, 439,598 SNPs were identified across 273,397 RADtags with an average coverage depth of 14.85x per sample. Due to low read coverage overall, only 506 variable SNPs were identified, 482 of which were from independent RADtags and could be used for population genetic analyses. Marker independence across sites was confirmed with no loci flagged as showing linkage disequilibrium. Although some loci showed significant deviation from Hardy-Weinberg, the loci involved differed across sites and were thus included in further analyses [45].

**Table 1:** Genomic summary statistics of populations sampled

Site	n	N alleles	θ	π	H_e_	H_o_
Northern Irish Sea^a^	6	2.50 ± 0.50	0.28 ± 0.14	132.56	0.53 ± 0.34	0.14 ± 0.19
North Atlantic^a^	8	2.45 ± 0.50	0.15 ± 0.08	73.08	0.5 ± 0.36	0.11 ± 0.16
Southern Irish Sea^a^	9	2.54 ± 0.50	0.26 ± 0.13	126.36	0.53 ± 0.28	0.01 ± 0.15
English Channel^a^	11	2.49 ± 0.50	0.29 ± 0.14	140.50	0.49 ± 0.33	0.09 ± 0.15
Bay of Biscay^a^	12	2.59 ± 0.49	0.28 ± 0.14	133.63	0.51 ± 0.32	0.11 ± 0.15
Tyrrhenian Sea	7	2.23 ± 0.42	0.22 ± 0.11	106.08	0.45 ± 0.36	0.08 ± 0.19
Balearic Sea	4	2.36 ± 0.48	0.29 ± 0.16	141.43	0.58 ± 0.31	0.11 ± 0.15^b^
Iberian Peninsula^a^	5	2.23 ± 0.42	0.26 ± 0.14	123.47	0.50 ± 0.34	0.09 ± 0.20
North Africa	6	2.53 ± 0.50	0.32 ± 0.16	152.42	0.55 ± 0.34	0.14 ± 0.19

Shown are site name, sample size for which usable DNA was available (n), mean number of alleles per locus (N alleles), nucleotide diversity (θ and π), expected heterozygosity (H_e_) and observed heterozygosity (H_o_). Standard deviations are indicated for mean values

^a^Temperature data available for this site; ^b^H_o_ significantly different to H_e_

All sites showed reasonable numbers of alleles per locus, nucleotide diversity and expected heterozygosity (N alleles = 2.44 ± 0.13, θ = 0.26 ± 0.05, H_e_ = 0.52 ± 0.04). Observed heterozygosity was low across all sites (H_o_ = 0.11 ± 0.02) but was significantly lower than H_e_ only in the Balearic Sea site, which had a low sample size (Table 1). Most molecular variance was found within rather than between sites (within = 71.42%, between = 28.58%, *P* < 0.01) and within rather than between the genetic clusters identified by STRUCTURE (see below) (within = 84.08%, between = 15.92%, *P* < 0.01).

**Fig. 2:**
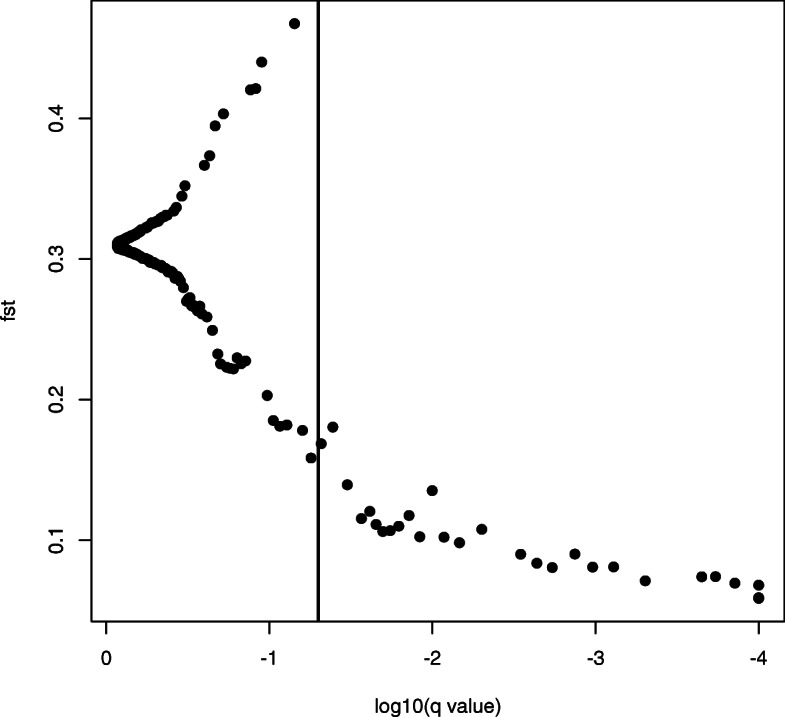
Posterior odds of the selection model and locus-specific F_ST_ for each SNP as calculated in BAYESCAN [46]. The vertical line represents the threshold for significance for a locus to be under selection of *P* ≥ 0.99. Loci to the right of the threshold line are suggested to be outlier loci

From the total number of SNPs, ARLEQUIN identified 191 as outliers (39.63%) and BAYESCAN identified 28 as outliers (Fig. 2; 5.81%). Twenty seven of the 28 outlier SNPs identified by BAYESCAN as outliers were also identified by ARLEQUIN. Therefore, the 27 outlier SNPs (5.60%) were removed from the data set and 455 (94.40%) were used in the neutral genetic analyses.

### Population structure

**Fig. 3:**
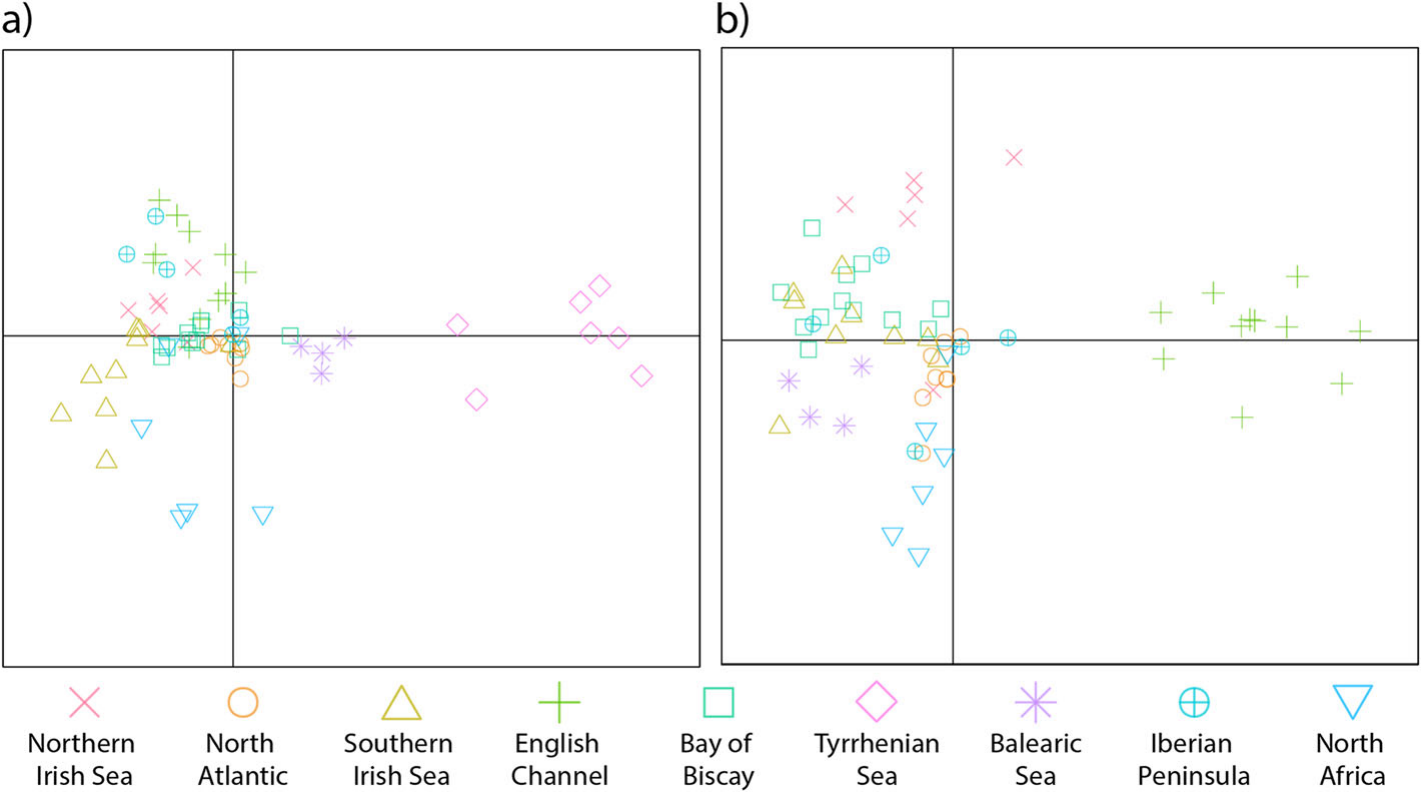
Principal component analysis using 455 neutral loci including **a** all sampling sites; and **b** hierarchical structure following removal of the highly differentiated site, Tyrrhenian Sea, as identified in a)

**Fig. 4:**
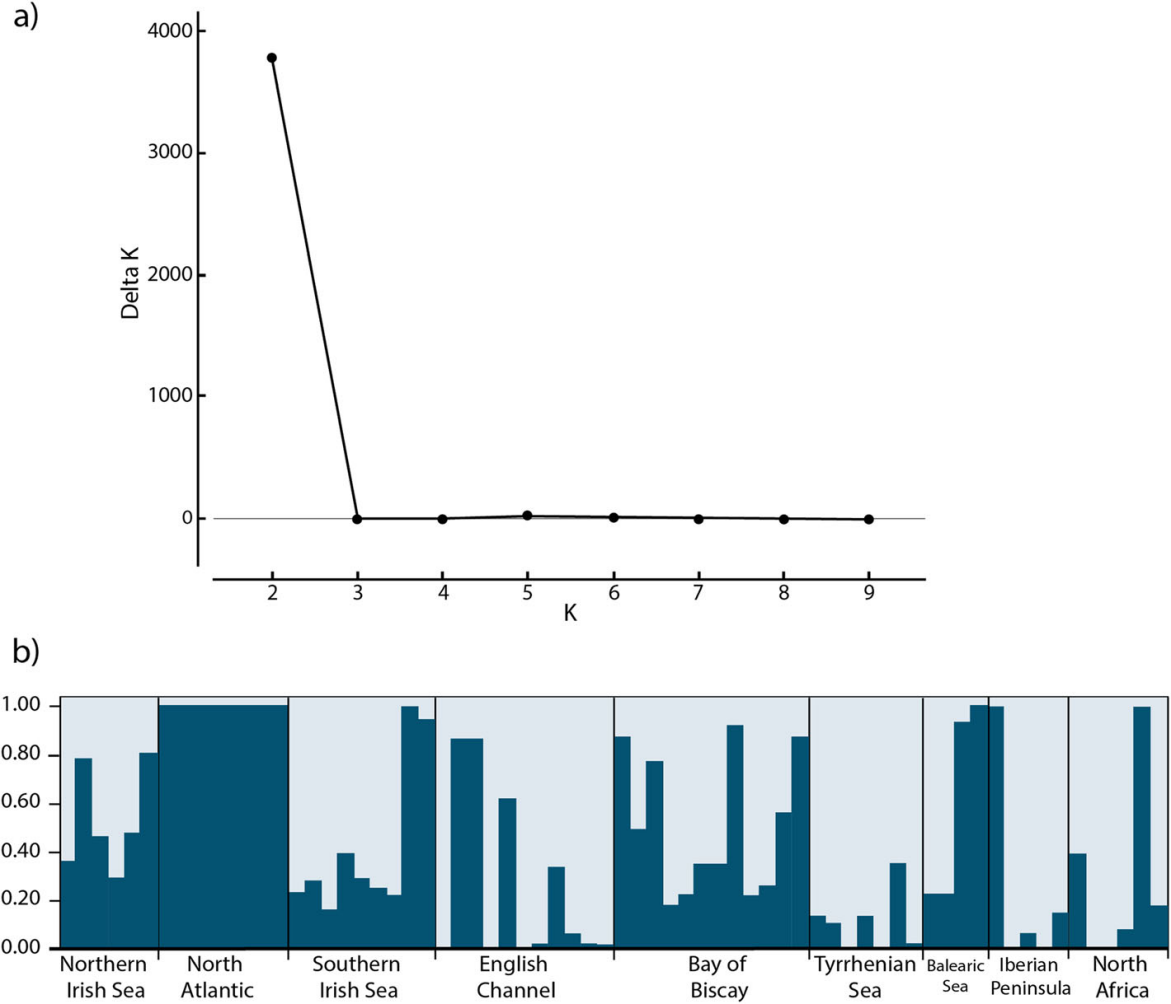
Population structure based on genetic clustering in STRUCTURE (Pritchard et al. 2000) using all loci. **a** ΔK for each value of K (putative number of populations), averaged over ten replicates. The peak at K = 2 shows the most likely number of genetic clusters within the sample. **b** STRUCTURE barplot for K = 2. Columns are individuals, with the proportion of an individual’s genotype assigned to each cluster (K) denoted by dark blue or light blue

**Fig. 5:**
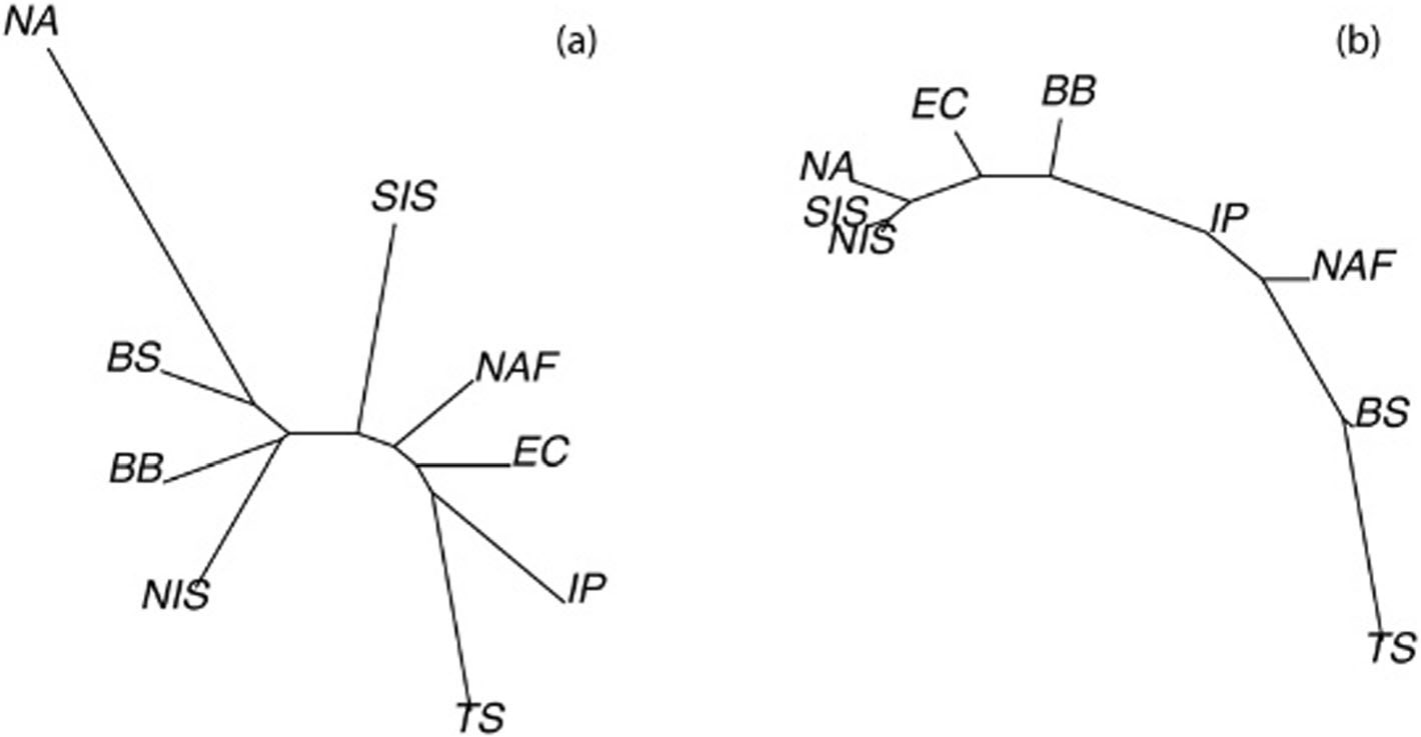
Neighbour-joining trees of **a** F_ST_ distances; **b** shortest coastline distances, among locations

Pairwise F_ST_ values for neutral loci had an average of 0.28 ± 0.10 across sites, with 25 out of the 36 comparisons showing significance following Bonferroni correction, strongly demonstrating a high degree of population substructure within the system as a whole (Table 2). In particular, the North Atlantic, Tyrrhenian Sea and Bay of Biscay sites were genetically differentiated from all other sites (Average F_ST_ = 0.42 ± 0.10; 0.32 ± 0.12 and 0.25 ± 0.05, respectively; Table 2), suggesting very low gene flow to and from these sites. The Discriminant Analysis of Principal Components (DAPC) results also showed the Tyrrhenian Sea to be highly differentiated from the other sites (Fig. 3a). When the Tyrrhenian Sea was excluded from the analysis, the English Channel population emerged as being differentiated from the remaining populations (Fig. 3b). Using the Bayesian cluster analysis in STRUCTURE, ΔK showed a clear peak at K = 2, suggesting two genetic clusters within the nine sites studied (Fig. 4a). Likelihood probability profiles also showed that K = 2 had the highest likelihood with low variance. The barplot in STRUCTURE for K = 2 identified that individuals from the North Atlantic site formed a distinct population from all the other sites combined (Fig. 4b). This result did not vary regardless of whether sampling location was included as a prior and there was no evidence of hierarchical clustering (K = 1 when North Atlantic individuals were removed). F_ST_ based isolation (Fig. 5a) by straight-line geographic distance (Table 2) and shortest ocean distance (Table 2; Fig. 5b) were not significant (*r* = 0.14, *P* = 0.27 and *r* = 0.08, *P* = 0.27, respectively).

**Table 2:** Pairwise genetic distances between sites (F_ST_; lower triangle) and coastline distances between sites (km; upper triangle)

	Northern Irish Sea	North Atlantic	Southern Irish Sea	English Channel	Bay of Biscay	Tyrrhenian Sea	Balearic Sea	Iberian Peninsula	North Africa
Northern Irish Sea	–	453	180	864	1286	4134	3075	1853	2447
North Atlantic	**0.36**	–	612	994	1332	3998	2939	1715	2311
Southern Irish Sea	**0.30**	**0.42**	–	768	1190	4041	2982	1760	2354
English Channel	**0.23**	**0.43**	**0.25**	–	834	3742	2683	1461	2056
Bay of Biscay	**0.18**	**0.31**	**0.25**	**0.20**	–	3390	2331	1109	1703
Tyrrhenian Sea	**0.35**	**0.58**	**0.31**	**0.20**	**0.31**	–	1177	2283	2150
Balearic Sea	0.22	**0.31**	0.25	0.22	**0.22**	**0.34**	–	1224	1091
Iberian Peninsula	0.32	**0.53**	**0.28**	0.19	**0.29**	**0.24**	0.27	–	597
North Africa	0.21	**0.44**	0.21	0.15	**0.22**	**0.22**	0.22	0.19	–

Numbers in bold are significant after Bonferroni correction (*p* < 0.0056)

**Fig. 6:**
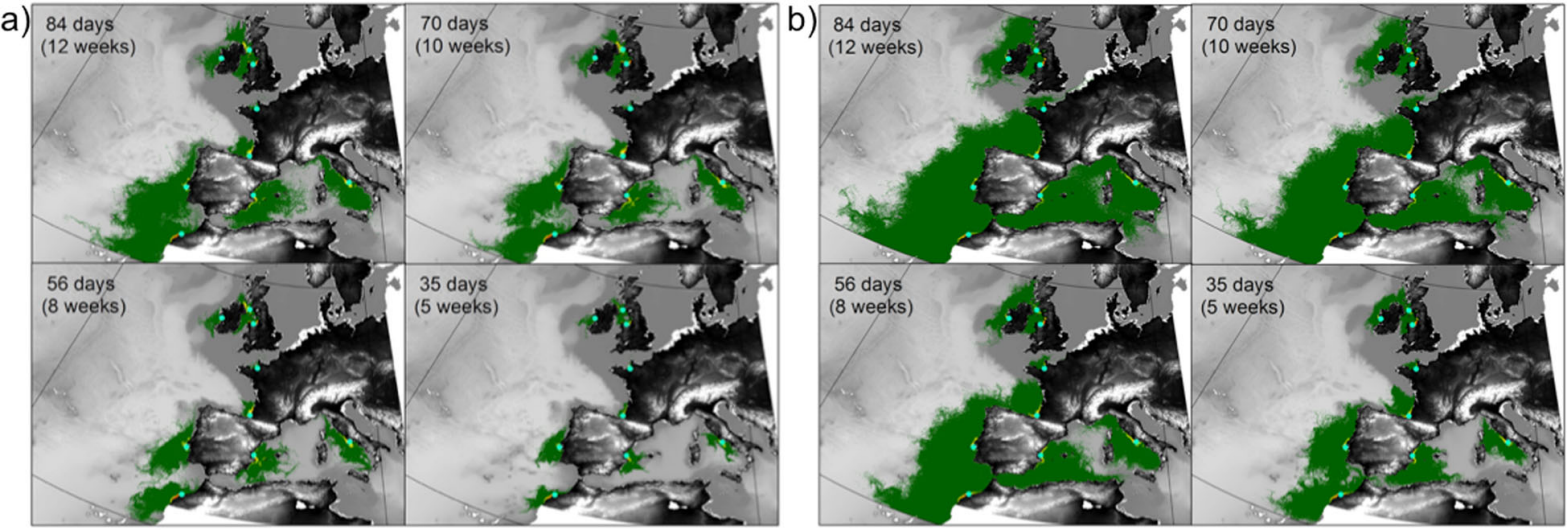
Maps of larval densities from dispersal simulations with **a** releases every 6 h over 1 week in April centred on the spring tide, representing the most conservative dispersal simulations; and **b** daily releases throughout each year of simulation, represent the most optimistic dispersal simulations. They show larval densities ranging between red (high), yellow (medium), and green (low). Maps, created in Matlab (Mathworks, Version R2016a), are shown for the four different planktonic larval durations ranging from 5 to 12 weeks

Maps of larval densities from dispersal simulations show the range of potential dispersal pathways for a single generation released from each sample location: from the most conservative (1 week of 6 hourly releases, 5 weeks maximum planktonic larval duration (PLD), Fig. 6a), to the most optimistic (daily releases all year round, 12 weeks PLD, Fig. 6b). The most optimistic dispersal scenarios are suggestive of isolation among sites (Additional file 1). Model simulations suggest isolation among the North Atlantic, northern Irish Sea, and southern Irish Sea sites (Additional file 1), which is reinforced by the F_ST_ distances (Table 2). Under the conservative scenario estimates, no larvae were exchanged among any sites in a single generation (Fig. 6a). Under the most optimistic scenario, high densities of larvae were retained close to natal release sites (average within 133 km, maximum 230 km) and very few larvae exchanged among release locations (Additional file 1). Potential connectivity was only identified between the northern Irish Sea and southern Irish Sea sites (as per [47]) but < 0.1% of the larval population made this transition. Dispersal distances away from source were shortest from the North Atlantic release site on the west coast of Ireland; this site exhibited the highest proportion of larval retention (79% self-recruited) and very few larvae reached nearby reefs, and none reached other release sites (Additional file 1). Dispersal resistance, as identified using ocean circulation modelling, did not show significant correlation with genetic distance, in terms of F_ST_, for any of the PLD scenarios (5 weeks: *r* = − 0.02, *P* = 0.59; 8 weeks: *r* = − 0.01, *P* = 0.59; 10 weeks: *r* = − 0.006, *P* = 0.57; 12 weeks: *r* = 0.00, *P* = 0.57).

### Loci under selection

Both ARLEQUIN and BAYESCAN analyses suggested that the 27 loci identified as outliers were under balancing selection (Fig. 2). Temperature data could not be collected from three of the study sites due to loss of equipment during the data collection period (Balearic Sea, Tyrrhenian Sea and North Africa). Temperature data was thus available for six of the study sites (Fig. 1) and only these study sites were included in the environmental association analyses. Of the temperature parameters collected from six of the sites (Table 3), no environmental associations were found with any loci.

**Table 3:** Temperature parameters per site

Temperature data	Temperature parameter	Northern Irish Sea	North Atlantic	Southern Irish Sea	English Channel	Bay of Biscay	Iberian Peninsula
All temperatures	Mean temp	10.82	11.62	11.93	14.21	16.67	16.10
	Max temp	37.85	39.37	34.78	41.54	42.13	35.48
	Min temp	−3.31	−5.62	−0.46	0.70	−2.46	3.32
	Temp range	41.16	44.99	35.24	40.84	44.59	32.16
	SD of mean	5.61	5.94	6.10	5.87	8.36	7.79
	95th – 5th percentile	14.38	15.40	15.77	23.68	21.74	18.59
	Average daily range	7.51	7.11	6.77	8.15	8.28	8.28
	Average daily SD	5.50	5.50	4.51	5.76	5.30	5.42
Water temperatures	Mean temp	10.55	11.53	11.76	14.00	16.76	15.75
	Max temp	16.14	19.73	17.24	22.50	25.09	21.17
	Min temp	4.52	3.67	5.88	5.91	7.90	11.18
	Temp range	11.62	16.06	11.36	16.59	17.19	9.99
Air temperatures	Mean temp	11.06	11.84	12.04	14.59	16.58	16.59
	Max temp	37.76	36.93	34.53	41.54	40.74	34.49
	Min temp	−2.28	−4.73	0.00	0.70	−1.03	3.53
	Temp range	40.04	41.66	34.53	40.84	41.77	30.96

Shown are all temperature data combined, water (high tide) temperatures and air (low tide) temperatures (°C). The mean, maximum, minimum, temperature range and average daily temperature range are shown for all temperatures, water temperatures and air temperatures, separately. The standard deviation (SD) of the mean, 95th – 5th percentile, and average daily standard deviation (SD) are also shown for all temperatures only

## Discussion

### Population structure

Population genetic analysis using genome-wide SNPs identified from nine *S. alveolata* reef sites spanning the latitudinal and longitudinal range of the species revealed low gene flow between sites, equating to low effective dispersal, with 25 out of 36 pairwise F_ST_ values showing significance and an average pairwise F_ST_ of 0.28 ± 0.10 across the system. The DAPC supported the differentiation of the Tyrrhenian Sea site from the other sites, as also identified by high F_ST_ values between this site and all other sites (F_ST_ = 0.32 ± 0.12), and the STRUCTURE analysis revealed the North Atlantic site to be a separate population from all the others (F_ST_ = 0.42 ± 0.10). Genetic divergence in terms of F_ST_ was not significantly correlated with either straight-line distance or shortest ocean distance. Presence of low gene flow between reef sites is in line with a growing number of studies that have identified population structuring in marine species which, as with *S. alveolata* in our study, have a dispersing larval stage and no hard geographical barriers to dispersal [17, 18, 48–50]. For instance, Benestan et al. (2015) [50] identified strong evidence of weak neutral genetic structure (average pairwise F_ST_ = 0.0019) in the American lobster (*H. americanus*) along a sea surface temperature gradient using RAD genotyping despite a dispersing planktonic larval stage with a duration of 4–6 weeks. Similarly, the bat star *Patiria miniate* (Brandt, 1835) showed genetic population structure along a latitudinal gradient (average pairwise F_ST_ = 0.061) despite a longer larval dispersal period of 6–10 weeks [49], which is in line with the estimated larval duration of *S. alveolata* at 5–12 weeks, dependant on temperature and food availability [42, 51]. Indeed, a meta-analysis revealed that mean pelagic larval duration was not a good predictor of gene flow [52] and our findings support this interpretation.

Three sites were differentiated from all other sites: North Atlantic (average pairwise F_ST_ = 0.42 ± 0.10 and STRUCTURE), Tyrrhenian Sea (average pairwise F_ST_ = 0.32 ± 0.12 and DAPC) and Bay of Biscay (average pairwise F_ST_ = 0.25 ± 0.05). The observed differences in the results produced by the two cluster assignment methods can be explained by how they use data: STRUCTURE assigns cluster groupings in order to minimise Hardy–Weinberg and linkage disequilibria within clusters by looking at allele frequencies [53, 54]. In contrast, DAPC maximizes the variance in linear combinations of allele frequencies (principal components) between groups while minimizing variance within groups [53]. Differences in genetic structure estimated by STRUCTURE and DAPC therefore likely result from how genetic variation is considered in either approach. The northern Irish Sea, southern Irish Sea (in terms of F_ST_) and English Channel (in terms of F_ST_ and the DAPC) sites were also all differentiated from each other (Table 2; Fig. 3b). The ocean circulation modelling predicted high isolation (zero to low larval density; Additional file 1) among all release sites, even in the most optimistic (dispersive) scenario. Therefore, the F_ST_ results are supported, at least in part, by patterns of ocean circulation influencing passive larval dispersal along the Atlantic and Mediterranean coastlines of Europe and North Africa.

High population sub-structuring could be partially explained by two behavioural barriers to connectivity: first, spawning in polychaetes is triggered by the action of waves, especially during spring tides, which tends to push larvae toward the coast rather than offshore [55] and second, *S. alveolata* larvae act to reduce dispersal out of the bay where they were spawned by moving higher in the water column with an incoming- and lower with an outgoing-tide [42]. This ‘tidal stream transport’, has been suggested as a possible mechanism for facilitating/restricting advective transport in a number of taxa (e.g. [56, 57]). Yet despite this, relatively high levels of genetic diversity were observed across the sites (H_e_ = 0.52 ± 0.04). Observed heterozygosity was very low at all sites and could be due to inbreeding [58], as a result of the site isolation predicted by the ocean circulation modelling and due to the reproductive strategy of gregarious colonial species, as juveniles settle together in patches and spawning of one individual triggers the spawning of those immediately adjacent [41]. Heterozygote deficiency has also been recorded in marine invertebrates as a consequence of the Wahlund effect, due to the coexistence of genetically distinct cohorts within a sampling site [18]. However, low heterozygosity estimations could also be a consequence of poor genome coverage and low sample size at some sites [59, 60]. Further research is needed to assess whether presence of null alleles is genome-wide, as evidence of inbreeding, or restricted to particular loci, as evidence of allelic dropout due to low coverage, in order to assess whether the species is at inbreeding risk.

Identifying the relative contribution of gene flow, genetic drift and natural selection to population structure is difficult in marine invertebrates due to their fluctuating population sizes [9]. This is particularly difficult in *S. alveolata*, as in-depth local ecological knowledge, such as population size and breeding behaviour, is lacking in the majority of sites (but see [33, 61–63]). That said, the identification by STRUCTURE of only the North Atlantic site as a separate, but not completely isolated, population, with all other sites forming a single population, supports the idea that low levels of gene flow are still maintaining genetic diversity within the system as a whole. It could be that asymmetric dispersal, a common feature of seascapes due to unidirectional currents [64] has led to some sharing of genotypes between the North Atlantic and other sites, as observed in the STRUCTURE plot (Fig. 4b). Such wide-ranging genetic connectivity has also been observed in other polychaete species [65]. This is potentially a positive sign for survival of the species in a changing climate, as the maintenance of genetic diversity is key to facilitating rapid evolution when environmental conditions change [66]. However, *S. alveolata* reduce their larval duration with increasing temperatures [42] and our models show that shorter larval duration leads to reduced larval dispersal. Therefore, lower connectivity, and thus gene flow, is predicted for *S. alveolata* in a changing climate.

Despite some evidence of low levels of gene flow between the majority of the study sites within the system, both F_ST_ values and STRUCTURE analysis revealed a level of isolation of the North Atlantic site and identified this reef as a separate population to all others. There are two possible processes that could, separately or in synergy, be causing the observed pattern of population structure: historical isolation and contemporary patterns of gene flow. During the Last Glacial Maximum, geomorphology and fossil evidence suggests that southwest Ireland was partially unglaciated [67] and genetic data supports the presence of a glacial refugium in this area for both terrestrial [68, 69] and marine species [70–72]. In particular, Jolly et al. (2006) [70] found that two coastal polychaete worms (*Lagis koreni* Malmgren, 1866, formerly *Pectinaria koreni,* and *Owenia fusiformis* Delle Chiaje, 1844) showed a private Irish Sea haplotype linking two ancestral haplotypes and they suggest this could have evolved in a small ice-free area along the southwest coast of Ireland. Therefore, one potential explanation is that this population was isolated in a different glacial refugium to the rest of the sites, with subsequent admixture, but further study is needed to test this hypothesis.

The second hypothesis, that contemporary gene flow is very low between the North Atlantic and other *S. alveolata* sites, is supported by predictions of larval dispersal as seen in the ocean circulation modelling. In even the most optimistic scenario for larval dispersal, the North Atlantic site was not predicted to have interchange of individuals with any of the other sampled sites and had by far the highest predicted proportion of larval retention (79% of released individuals were retained compared to an average of 20 ± 13% for all other sites). This can be explained by the hydrodynamic modelling in terms of current patterns within Galway Bay limiting dispersal. The presence of the North Atlantic Current, which moves eastwardly towards Ireland and then continues northwards, is also an isolating factor for the North Atlantic reef. Any larvae that do move beyond their spawning reef at the North Atlantic site are drawn northwards, beyond the current northern range limit for the species (the Solway Firth, Scotland [43]). This oceanic barrier is also a likely cause of the observed genetic divergence between the northern/southern Irish Sea sites and the English Channel, and the reason that the dispersing larvae from more southerly sites do not reach the North Atlantic site. Although larval dispersal of many other marine species has been found to be mediated by the North Atlantic Current (e.g. [73, 74]), isolation due to the North Atlantic Current has not previously been reported, likely because it depends upon species-specific occurrence range and life history traits. Therefore, reduced contemporary gene flow is a likely cause of the observed population isolation of the North Atlantic *S. alveolata* reef. These findings highlight the North Atlantic reef site as at risk if population size reductions occur as recruitment and genetic augmentation is unlikely from elsewhere within the species’ range. Therefore, conservation management is needed to ensure that population size does not decrease at this site, and potentially throughout Ireland.

Despite the importance of ocean circulation to larval dispersal in general [18, 19, 75] and to *S. alveolata* in particular, as identified by our data in regards to the isolated North Atlantic site, our ocean circulation models of larval dispersal did not show a significant correlation with observed genetic distance between sites. This is surprising as both F_ST_ values and larval dispersal models suggest low connectivity between sites. This lack of correlation between our larval dispersal modelling and genetic distance values is in contrast to studies on a number of species with a dispersing larval stage including the Mediterranean shore crab (*Carcinus aestuarii* Nardo, 1847) [19], the bat star (*P. miniata*) [49] and the American lobster (*H. americanus*) [17]; these studies found genetic structure was directly related to ocean currents or to estimates of potential larval connectivity obtained with coupled physical-biological models. However, these studies were conducted over a smaller geographical area than our study. As per our study, Jorde et al. (2015) [76] found that when looking at large-scale differentiation patterns in the north Atlantic, geographic distance and larval drift alone explained only a minor portion (2.5–4.7%) of genetic isolation in the northern shrimp (*Pandalus borealis* Krøyer, 1838). Galindo et al. (2010) [48] modified a biophysical model for Monterey Bay in California to simulate dispersal of the acorn barnacle (*Balanus glandula* Darwin, 1854) but it also did not match an observed genetic cline in the species. They discovered that their model fit was improved by including natural selection, larval retention, and input values from an additional source population [48].

There are several factors likely to reduce the similarity between modelled larval dispersal and observed gene flow. Gene flow is representative of multigenerational mixing, while the model used in this study is representative of the dispersal patterns of only one generation. It is possible to simulate multigenerational gene flow based on dispersal models [49, 77, 78] but this would be confounded by the lack of known sites that could be used as “stepping stone” sites, which may facilitate dispersive spread over multiple generations. Such sites are likely to exist but are currently undocumented. These undiscovered sites represent missing source populations within our model and may well be a cause of the observed mismatch between predicted larval dispersal and observed gene flow. The distribution and occurrence of *S. alveolata* reefs are not well documented, particularly in the southern range of the species (Firth, unpublished), and the prediction of our model that genetic differentiation between our sample sites would be high could be due to the absence of intermediate reef sites within the model that would create higher levels of admixture within the system as a whole [9, 79]. This is further complicated by the fact that reefs can vary temporally in their presence at a site [62]. Therefore, increased knowledge of the location of *S. alveolata* reefs and ongoing monitoring of reef sites is required to generate a clearer picture of the role of connectivity in meta-population maintenance in this species and to further inform the ocean circulation model. Our study highlights the importance of comparing bio-physical models with observed population structure of a species in order to create accurate dispersal predictions.

### Loci under selection

Twenty-seven SNPs were identified as outlier loci, all of which were putatively under balancing selection. Identification of a higher number of loci potentially under balancing rather than divergent selection is common in genome scan studies, including those on marine invertebrates [12, 50, 80–82]. Our finding that 5.6% of SNPs were potentially under balancing selection is reflective of a growing body of evidence that suggests that balancing selection, which acts to preserve polymorphism, is more important in the genome than previously considered [12, 50, 83]. In their study of the sea anemone, *Nematostella vectensis* Stephenson, 1935, covering a broad geographical range, Reitzel et al. (2013) [12] identified 37 polymorphic sites inferred to be under balancing selection, but none under divergent selection, as with our study. However, further evidence in the form of elevated polymorphism, reduced differentiation, and shifts towards intermediate allele frequencies are needed to confirm that these loci are indeed under balancing selection [58].

The lack of identification of loci under divergent selection using either outlier analyses or environmental association tests is surprising given that local adaptation to temperature is known to be present in this system [43]. We previously reported that *S. alveolata* individuals from sites along the latitudinal range of this species (all of which are included in this study) showed different responses to thermal regime changes in terms of their membrane lipid composition dependant on their site of origin [43]. This is likely a reflection of poor genome coverage leading to a low number of genotyped loci (as per [12]) and whole genome sequencing would be beneficial to search for adaptive loci and their functions in this system [84].

## Conclusions

Modelling of larval dispersal predicted low gene flow between sites for *S. alveolata* across their range, which was in part supported by the genomic data. In particular, population genetic analyses identified the North Atlantic reef is an isolated population, due to the effect on dispersal of the North Atlantic Current. These findings have implications for the management of *S. alveolata* engineered habitats in a changing climate and have highlighted the North Atlantic reef as at increased risk and in need of direct conservation action in order to preserve not only this species, but above all the crucial functional ecological roles (biodiversity hotspots, coastal protection, carbonate traps, etc.) that are associated with these bioconstructions. Our study highlights the utility of using seascape genomics to identify populations of conservation concern.

## Methods

### Sampling and molecular methods

In total, 180 mature individual worms were haphazardly collected from nine sites (20 per site) across the global geographic range between August 2013 and March 2014, from North Africa at the species’ southern boundary, along the Atlantic Coast, to the Northern Irish Sea in the north, as well as from two sites on the Mediterranean coast (Fig. 1). Individuals were placed immediately in RNA-later (Qiagen Inc., Crawley), maintained at room temperature for between 48 h and 1 week and then stored at − 20 °C until DNA extraction.

Genomic DNA was extracted using chaotropic “Chaos” buffer (4 M guanidine thiocyanate; 0.5% N-lauroyl sarcosine; 25 μM tris(hydroxymethyl)aminomethane (Tris), pH 8.0; 0.1 M 2-mercaptoethanol, 0.5% N-lauroyl sarcosine) (modified from Fukami et al. 2004 [85]). Worms were homogenised within the buffer at room temperature for at least 48 h before adding an equal volume of phenol extraction buffer (PEB) and double the volume of phenol:chloroform:isoamyl alcohol (25:24:1). From here the protocol followed a phenol-chloroform extraction with isopropanol precipitation [86]. An additional ammonium acetate (3 M) precipitation step was added to further purify DNA. DNA was resuspended in sterile distilled H_2_O, quality assessed using a NanoDrop 8000 spectrophotometer (Thermo Scientific, Wilmington, DE), visualised on 1% agarose gels, and quantified using the Quant-IT PicoGreen dsDNA Assay Kit (Life Technologies, Carlsbad, California). Single-end Restriction-site Associated DNA (RAD) libraries were prepared by digesting DNA from each individual using the EcoRI and MseI restriction enzymes. Samples were individually barcoded and sequenced (Ecogenics, Zurich-Schlieren, Switzerland) using an Illumina NextSeq 500 (Illumina, San Diego, California).

### SNP detection and summary statistics

All analyses were carried out on the Analyses and Bioinformatics for Marine Science (ABiMS) Galaxy Platform at the Station Biologique de Roscoff (galaxy.sb-roscoff.fr [87];). Samples with mean Phred scores of < 25 or read coverage of < 150,000 were removed from the dataset and sequences were trimmed to 60 bp length using Trimmomatic version 0.36.4 [88]. STACKS version 1.46.0 [89] was used to de novo map the sequences and call SNPs by sequentially running “ustacks”, “cstacks” and “sstacks” using the parameters suggested to be optimal for population genetic inference by [90] namely, allowing a minimum depth of coverage of three (ustacks: m; stack coverage), a maximum of two mismatches between reads for a single individual (ustacks: M), and four mismatches between primary and secondary reads (ustacks: N). These settings have been found to increase the number of loci and reduce the SNP and allele error rates within a dataset [90]. Furthermore, the low value of M used here reduces the risk of ﻿paralogous loci being merged into the same SNP [91]. The number of mismatches allowed between loci was set at three (cstacks: n [91];).The removal or break up of highly repetitive RAD tags was permitted within the program (ustacks: deleverage).

The programme *populations* from the STACKS suite, was used to process the SNP data [89]. RADtags with at least 10x depth of read coverage were retained to ensure accuracy of heterozygous SNP calls (populations: p [50];). Lumped paralogs can be a concern in high coverage data sets [92], but 10x coverage is considered low [93] and thus reduces this to a low risk factor. A locus had to be present in 70% of the populations to be included in the analysis, which allows for mutations in restriction sites that may cause loci to dropout in certain lineages [50, 94] and the minor allele frequency within populations (populations: min_maf) was set at > 0.01 [91]. In order to maximise SNP discovery, the percentage of individuals required within a population to process a locus was set at 50% (populations: r). Only a single SNP per RADtag was retained for subsequent analyses to avoid problems of non-independence between markers [10]. The STRUCTURE file output by *populations* was transformed in PGD Spider version 2.1.0.3 [95] for use in all further analyses. Each population and locus was tested for deviation from Hardy-Weinberg equilibrium and linkage disequilibrium in ARLEQUIN version 3.5.2.2 [96] and significance was assessed following Bonferroni correction for multiple tests. Loci that showed null alleles in multiple populations, or with significant deviation from Hardy–Weinberg equilibrium, were removed from further analyses ﻿to reduce the risk of inclusion of paralogous sequence variants [97].

Genomic summary statistics were calculated in ARLEQUIN version 3.5.2.2 [96] as mean number of alleles per locus (N alleles), nucleotide diversity (θ and π), expected heterozygosity (H_e_) and observed heterozygosity (H_o_). Separate analyses of molecular variance (AMOVA) were carried out based on site of origin and genetic clusters identified in STRUCTURE (see below) using 16,000 permutations.

An outlier approach was used to identify loci that had a higher or lower F_ST_ than expected under a neutral model of selection (under positive or balancing selection, respectively). Outliers were estimated in two ways: firstly, using BAYESCAN version 2.1 [46], which uses a Bayesian approach to estimate the posterior probability that a locus is affected by selection, and was run using 20 pilot runs of 5000 iterations each, a total of 1,050,000 iterations (sample size of 100,000 and a thinning interval of 10), and a burn-in of 50,000. Only loci with a posterior probability (P) ≥ 0.95 with a prior odd of 10 were considered as outliers. BAYESCAN has consistently outperformed other outlier detection methods in terms of lack of false positives [4, 17, 98]. Outliers were also calculated in ARLEQUIN using 100,000 simulations, 500 demes, and a maximum expected heterozygosity of 0.5 [99]. Outlier SNPs were identified using a *P*-value of ≤0.05. Only SNPs identified as outliers by both BAYESCAN and ARLEQUIN were conservatively selected as candidate loci under balancing or directional selection. Subsequent analyses of population structure and seascape genomics were conducted separately for neutral and outlier loci.

### Population structure

Pairwise F_ST_ were calculated in ARLEQUIN version 3.5.2.2 [96] and significance in certainty of the estimator assessed following Bonferroni correction for multiple tests. Straight-line distances between sites were calculated using ArcGIS Version 10.1 (ESRI, 2011) and shortest ocean distances between sites were calculated in R version 3.1.2 [44] using the package *marmap* [100], where distance was calculated excluding positive elevation [99]. Isolation-by-distance (straight line and shortest ocean) was tested in ARLEQUIN (Version 3.5.2.2) using a Mantel test [101] with 10,000 permutations. To visualise these relationships, neighbor-joining (nj) tree estimation plots were constructed in R package *ape 5.3* [102], following the methods outlined in [103], and were constructed using F_ST_ and shortest ocean distances for each pairwise (site) combination. Divergence between sites was also assessed using a Discriminant Analysis of Principal Components (DAPC) with the R package *adegenet 2.1.2* [104], with 30 components and two discriminant functions retained in the analysis. Hierarchical structure was also considered by carrying out a second DAPC excluding the most divergent site, as identified in the first DAPC.

Genetic clusters (populations) were inferred using Bayesian analyses carried out in STRUCTURE version 2.3.4 [54]. The analyses assumed admixture, correlated allele frequencies, and were run with 100,000 burn-in cycles and 100,000 Markov Chain Monte Carlo runs. The number of populations (K) was considered between 1 and 10, with 10 replicates per K. This was repeated with and without location priors, and using a hierarchical approach to resolve fine-scale genetic structure by removing highly differentiated populations [45]. The most likely value of K was inferred by estimating ΔK using the Evanno method [105], as implemented in Structure Harvester version 0.6.94 [106].

Hydrodynamic models are increasingly used as predictors of larval dispersal (gene flow) in marine systems (e.g. [20, 107, 108] and many others). Probabilistic dispersal simulations of potential larval connectivity were run using the Connectivity Modeling System (CMS, [109]) paired with 3-hrly Hybrid Coordinate Ocean Model (HYCOM) and Navy coupled ocean data assimilation (NCODA) global 1/12° daily outputs from 2004, 2010, and 2012 [110]. These years were selected to represent neutral, negative and positive phases of the North Atlantic Oscillation (NAO) respectively, and are thus likely to reflect average dispersal patterns over the last decade [109, 111]. Vertical velocities were calculated using the Eulerian continuity equation, and a random diffusive kick was added at each hourly dispersal time-step to better capture the potential effects of sub-gridscale turbulent diffusion (horizontal = 15 m^2^s^− 1^, vertical = 0.05 m^2^s^− 1^; after Kough et al. 2016 [112]). HYCOM is a global model appropriate for large landscape continental-scale dispersal simulations, but does not include tides. Larvae were considered as passive particles even though diel vertical migrations may occur [42]. Simulations can therefore be over-dispersive, as the inclusion of tides and behaviour is liable to be retentive [113].

In the simulations, 100 propagules, each representing an *S. alveolata* larva, were released in a Lagrangian framework (so that each theoretical larvae was tracked individually), from each of nine locations (Fig. 1), and their dispersal under two different release scenarios predicted: (1) a *conservative* scenario with propagule releases every 6-h over 1 week in April for each year, centred on the spring tide, and (2) an *optimistic* scenario which modelled a daily release on every day of the year. The *optimistic* scenario was designed to simulate all the potential pathways of dispersal if reproduction occurs throughout the year, which has been suggested in *S. alveolata* [42], thus capturing the full variability of potential larval fates. Larval competency to settle was assumed after 4 weeks, and a maximum PLD of 12 weeks applied based on field and laboratory estimates [42, 51]. Larvae were permitted to settle when located within 5 km of a known reef site (Additional file 1). Additional (non-release) sites were also included as reported in The Global Biodiversity Information Facility (GBIF.org) in which larvae could also settle if encountered. Larval exchange counts (i.e. the source-sink contribution) were generated after 5, 8, 10, and 12 weeks PLD. Model outputs were processed in Matlab (Mathworks, Version R2016a). Pairwise matrices of larval exchange counts, standardised using proportions, were generated from larval fates averaged across the three focus years to compare against genetic pairwise F_ST_ matrices. Daily positions of larvae throughout the simulation were used to generate cumulative 2-D maps of larval density to highlight the predicted pathways of dispersal and areas of potential settlement.

The proportion of settlers reaching each study site from each release site under the four larval duration scenarios (5, 8, 10 and 12-weeks) in the ocean circulation modelling simulations were inverted and used to form a matrix of pairwise dispersal resistances (i.e. the proportion of larvae that did not reach each site). Mantel tests [101] were carried out in ARLEQUIN (Version 3.5.2.2) [96] using 16,000 permutations to assess the correlation between pairwise F_ST_ (using putatively neutral loci) and dispersal. A Bonferroni correction was applied to assess the significance of the Mantel tests [114].

### Loci under selection

Locally collected temperature data provides insight into the conditions that *S. alveolata* experience at a spatial and temporal scale that cannot be achieved using satellite data [115] and can be used for comparison with genomic data, to test genotype-environment associations [116]. Temperature data were obtained from Seabra et al. (2015) [117] who used biomimetic temperature loggers placed in the intertidal zone of exposed shores [115, 118]. At five sites, temperature data were collected with a resolution of 0.5 °C at mid-intertidal level every hour between July 2010 and July 2014. High- and low-tide temperatures were also identified to the nearest hour. At the remaining four sites (i.e. English Channel, Balearic Sea, Tyrrhenian Sea and North Africa), an iButton (Maxim, Munich) was placed in the mid-littoral zone, collecting temperature data with a resolution of 0.5 °C every 3 h between April 2014 and April 2015. Temperature readings closest to high- and low-tide were identified.

*S. alveolata* experience tidally-driven fluctuations in water and air temperatures. Sixteen metrics were calculated to describe variation in temperature at each site as follows: mean temperature, maximum temperature, minimum temperature, temperature range (maximum-minimum), standard deviation of the mean, 95th – 5th percentile (to exclude outlier temperatures), average daily temperature range (maximum-minimum per day), and average daily standard distribution (standard deviation of the mean), per site. High-tide (water) and low-tide (air) temperature values were also used separately to calculate mean temperature, maximum temperature, minimum temperature, and temperature range (maximum-minimum) for water and air temperatures, respectively (Table 3). Larvae of *S. alveolata* can be found in the water column all year round [42], suggesting that they are reproductively active throughout the year, thus temperature data from all months were included in the analyses.

We utilised a gene-environment association software, BAYENV2 [119], to test for covariance between SNP allele frequencies and the 16 thermal variables. BAYENV2 controls for neutral population structure by first estimating a covariance matrix among populations for all loci and then accounting for that covariance in the gene-environment association test [120, 121]. The programme was run with 100,000 iterations for both neutral parameterisation and association testing [122]. Outlier loci were classed as those with a Bayes Factor > 3 [8, 123, 124].

## Supplementary information

**Additional file 1.** SeascapeGenomics_Muiretal.

## Data Availability

DNA sequences are available on NCBI SRA (http://www.ncbi.nlm.nih.gov/bioproject/645800; BioSample accessions: SAMN15516880–978) [125]. Sample site locations, temperature data and ocean circulation model outputs are available on FIGSHARE (https://figshare.com/articles/dataset/Seascape_genomics_reveals_population_isolation_in_the_reef-building_honeycomb_worm_Sabellaria_alveolata_L_/12645896) [126]. Galaxy workflow is available on github: https://github.com/annamuir/sabellaria.git

## Abbreviations

PLD: Planktonic larval duration
RADseq: Restriction-site associated DNA sequencing

## References

[1] Reusch TBH, Wood TE (2007). Molecular ecology of global change. Mol Ecol..

[2] Holderegger R, Kamm U, Gugerli F (2006). Adaptive vs. neutral genetic diversity: implications for landscape genetics. Landsc Ecol..

[3] Dionne M, Caron F, Dodson JJ, Bernatchez L (2008). Landscape genetics and hierarchical genetic structure in Atlantic salmon: the interaction of gene flow and local adaptation. Mol Ecol..

[4] Chávez-Galarza J, Henriques D, Johnston JS, Azevedo JC, Patton JC, Muñoz I, De La Rúa P, Pinto MA (2013). Signatures of selection in the Iberian honey bee (*Apis mellifera iberiensis*) revealed by a genome scan analysis of single nucleotide polymorphisms. Mol Ecol..

[5] Matala AP, Ackerman MW, Campbell MR, Narum SR (2014). Relative contributions of neutral and non-neutral genetic differentiation to inform conservation of steelhead trout across highly variable landscapes. Evol Appl..

[6] Conover DO, Schultz ET (1995). Phenotypic similarity and the evolutionary significance of countergradient variation. Trends Ecol Evol..

[7] Merilä J, Hendry AP (2014). Climate change, adaptation, and phenotypic plasticity: the problem and the evidence. Evol Appl..

[8] Pujolar JM, Jacobsen MW, Als TD, Frydenberg J, Munch K, Jõnsson B, Jian JB, Cheng L, Maes GE, Bernatchez L, Hansen MM (2014). Genome-wide single-generation signatures of local selection in the panmictic European eel. Mol Ecol..

[9] Gleason LU, Burton RS (2016). Genomic evidence for ecological divergence against a background of population homogeneity in the marine snail *Chlorostoma funebralis*. Mol Ecol..

[10] Lemay MA, Russello MA (2015). Genetic evidence for ecological divergence in kokanee salmon. Mol Ecol..

[11] Hohenlohe PA, Bassham S, Etter PD, Stiffler N, Johnson EA, Cresko WA (2010). Population genomics of parallel adaptation in threespine stickleback using sequenced RAD tags. PLoS Genet..

[12] Reitzel AM, Herrera S, Layden MJ, Martindale MQ, Shank TM (2013). Going where traditional markers have not gone before: utility of and promise for RAD sequencing in marine invertebrate phylogeography and population genomics. Mol Ecol..

[13] Sandoval-Castillo J, Robinson NA, Hart AM, Strain LWS, Beheregaray LB (2018). Seascape genomics reveals adaptive divergence in a connected and commercially important mollusc, the greenlip abalone (*Haliotis laevigata*), along a longitudinal environmental gradient. Mol Ecol..

[14] Wernberg T, Smale DA, Thomsen MS (2012). A decade of climate change experiments on marine organisms: procedures, patterns and problems. Glob Chang Biol..

[15] Munday PL, Warner RR, Monro K, Pandolfi JM, Marshall DJ (2013). Predicting evolutionary responses to climate change in the sea. Ecol Lett..

[16] Reusch TBH (2014). Climate change in the oceans: evolutionary versus phenotypically plastic responses of marine animals and plants. Evol Appl..

[17] Benestan L, Quinn BK, Maaroufi H, Laporte M, Clark FK, Greenwood SJ, Rochette R, Bernatchez L, Clark FK, Greenwood SJ, Rochette R, Bernatchez L (2016). Seascape genomics provides evidence for thermal adaptation and current-mediated population structure in American lobster (*Homarus americanus*). Mol Ecol..

[18] Coscia I, Robins PE, Porter JS, Malham SK, Ironside JE (2013). Modelled larval dispersal and measured gene flow: seascape genetics of the common cockle *Cerastoderma edule* in the southern Irish Sea. Conserv Genet..

[19] Schiavina M, Marino IAM, Zane L, Melià P (2014). Matching oceanography and genetics at the basin scale. Seascape connectivity of the Mediterranean shore crab in the Adriatic Sea. Mol Ecol..

[20] Xuereb A, Benestan L, Normandeau É, Daigle RM, Curtis JMR, Bernatchez L, Fortin MJ (2018). Asymmetric oceanographic processes mediate connectivity and population genetic structure, as revealed by RADseq, in a highly dispersive marine invertebrate (*Parastichopus californicus*). Mol Ecol..

[21] Helmuth B, Mieszkowska N, Moore P, Hawkins SJ (2006). Living on the edge of two changing worlds: forecasting the responses of rocky intertidal ecosystems to climate change. Annu Rev Ecol Evol Syst..

[22] Harley CDG (2011). Climate change, keystone predation, and biodiversity loss. Science..

[23] Marshall DJ, Monro K, Bode M, Keough MJ, Swearer S (2010). Phenotype-environment mismatches reduce connectivity in the sea. Ecol Lett..

[24] Jones C, Lawton J, Shachak M, Samson F, Knopf F (1996). Organisms as Ecosystem Engineers. Ecosystem Management.

[25] Chaverra A, Wieters E, Foggo A, Knights AM (2019). Removal of intertidal grazers by human harvesting leads to alteration of species interactions, community structure and resilience to climate change. Mar Environ Res..

[26] Gruet Y (1986). Spatio-temporal changes of sabellarian reefs built by the sedentary polychaete *Sabellaria alveolata*. Mar Ecol..

[27] Wilson DP (1968). The settlement behaviour of the larvae of *Sabellaria alveolata* (L.). J Mar Biol Assoc U K..

[28] Wilson DP (1970). Additional observations on larval growth and settlement of *Sabellaria alveolata*. J Mar Biol Assoc UK..

[29] Buffet JP, Corre E, Duvernois-Berthet E, Fournier J, Lopez PJ (2018). Adhesive gland transcriptomics uncovers a diversity of genes involved in glue formation in marine tube-building polychaetes. Acta Biomater..

[30] Fournier J, Etienne S, Le Cam J-B (2010). Inter- and intraspecific variability in the chemical composition of the mineral phase of cements from several tube-building polychaetes. Geobios..

[31] Dubois SF, Commito JA, Olivier F, Retière C (2006). Effects of epibionts on Sabellaria alveolata (L.) biogenic reefs and their associated fauna in the bay of Mont saint-Michel. Estuar Coast Shelf Sci.

[32] Noernberg MA, Fournier J, Dubois SF, Populus J (2010). Using airborne laser altimetry to estimate *Sabellaria alveolata* (Polychaeta: Sabellariidae) reefs volume in tidal flat environments Mauricio. Estuar Coast Shelf Sci..

[33] Jones AG, Dubois SF, Desroy N, Fournier J (2018). Interplay between abiotic factors and species assemblages mediated by the ecosystem engineer *Sabellaria alveolata* (Annelida: Polychaeta). Estuar Coast Shelf Sci..

[34] Desroy N, Dubois SF, Fournier J, Ricquiers L, Le Mao P, Guerin L, Gerla D, Rougerie M, Legendre A (2011). The conservation status of *Sabellaria alveolata* (L.) (Polychaeta: Sabellariidae) reefs in the bay of mont-Saint-Michel. Aquat Conserv Mar Freshwat Ecosyst..

[35] Dubois SF, Barillé L, Cognie B (2009). Feeding response of the polychaete *Sabellaria alveolata* (Sabellariidae) to changes in seston concentration. J Exp Mar Biol Ecol..

[36] Porras R, Bataller JV, Murgui E, Torregrosa MT (1996). Trophic structure and community composition of polychaetes inhabiting some *Sabellaria alveolata* (L.) reefs along the Valencia Gulf Coast, Western Mediterranean. Mar Ecol..

[37] Dubois SF, Retière C, Olivier F (2002). Biodiversity associated with *Sabellaria alveolata* (Polychaeta: Sabellariidae) reefs: effects of human disturbances. J Mar Biol Assoc U K..

[38] De Smet B, D’Hondt AS, Verhelst P, Fournier J, Godet L, Desroy N, Rabaut M, Vincx M, Vanaverbeke J (2015). Biogenic reefs affect multiple components of intertidal soft-bottom benthic assemblages: the *Lanice conchilega* case study. Estuar Coast Shelf Sci..

[39] Farcy S. Analyse de la structure génétique des récifs de *Sabellaria alveolata* (L.) dans la baie du Mont-Saint-Michel à l’aide de marqueurs microsatellites (masters thesis): Université Pierre et Marie Curie – Paris VI; 2003.

[40] Rigal F. Barrières biogéographiques et processus historiques chez les invertébrés marins : définition des unités taxonomiques et populationnelles chez *Sabellaria alveolata* (masters thesis): Université de Paris XI; 2005.

[41] Pawlik JR (1988). Larval settlement and metamorphosis of two gregarious Sabellariid polychaetes: *Sabellaria alveolata* compared with *Phragmatopoma californica*. J Mar Biol Assoc U K..

[42] Dubois SF, Comtet T, Retière C, Thiébaut E (2007). Distribution and retention of *Sabellaria alveolata* larvae (Polychaeta: Sabellariidae) in the bay of Mont-saint-Michel. France. Mar Ecol Prog Ser..

[43] Muir AP, Nunes FLD, Dubois SF, Pernet F (2016). Lipid remodelling in the reef-building honeycomb worm, Sabellaria alveolata, reflects acclimation and local adaptation to temperature. Sci Rep..

[44] Team RC (2014). R: A Language and Environment for Statistical Computing. R Foundation for Statistical Computing Vienna, Austria.

[45] Muir AP, Thomas R, Biek R, Mable BK (2013). Using genetic variation to infer associations with climate in the common frog, *Rana temporaria*. Mol Ecol.

[46] Foll M, Gaggiotti O (2008). A genome-scan method to identify selected loci appropriate for both dominant and codominant markers: a Bayesian perspective. Genetics..

[47] Robins PE, Neill SP, Giménez L, Stuart R, Jenkins SR, Malham SK (2013). Physical and biological controls on larval dispersal and connectivity in a highly energetic shelf sea. Limnol Oceanogr..

[48] Galindo HM, Pfeiffer-Herbert AS, McManus MA, Chao Y, Chai F, Palumbi SR (2010). Seascape genetics along a steep cline: using genetic patterns to test predictions of marine larval dispersal. Mol Ecol..

[49] Sunday JM, Popovic I, Palen WJ, Foreman MGG, Hart MW (2014). Ocean circulation model predicts high genetic structure observed in a long-lived pelagic developer. Mol Ecol..

[50] Benestan L, Gosselin T, Perrier C, Sainte-Marie B, Rochette R, Bernatchez L (2015). RAD genotyping reveals fine-scale genetic structuring and provides powerful population assignment in a widely distributed marine species, the American lobster (*Homarus americanus*). Mol Ecol..

[51] Cazaux C (1964). Developement larvaire de *Sabellaria alveolata*. Oceanography Monaco..

[52] Weersing K, Toonen RJ (2009). Population genetics, larval dispersal, and connectivity in marine systems. Mar Ecol Prog Ser..

[53] Jombart T, Devillard S, Balloux F (2010). Discriminant analysis of principal components: a new method for the analysis of genetically structured populations. BMC Genet..

[54] Pritchard JK, Stephens M, Donnelly P (2000). Inference of population structure using multilocus genotype data. Genet Mol Res..

[55] McCarthy DA, Young CM, Emson RH (2003). Influence of wave-induced disturbance on seasonal spawning patterns in the sabellariid polychaete *Phragmatopoma lapidosa*. Mar Ecol Prog Ser..

[56] Tankersley RA, Forward RB (1994). Endogenous swimming rhythms in estuarine crab megalopae: implications for flood-tide transport. Mar Biol..

[57] Knights AM, Crowe TP, Burnell G (2006). Mechanisms of larval transport: vertical distribution of bivalve larvae varies with tidal conditions. Mar Ecol Prog Ser..

[58] Buckley J, Holub EB, Koch MA, Vergeer P, Mable BK (2018). Restriction associated DNA-genotyping at multiple spatial scales in *Arabidopsis lyrata* reveals signatures of pathogen-mediated selection. BMC Genomics..

[59] Benestan L, Ferchaud AL, Hohenlohe PA, Garner BA, Naylor GJP, Baums IB, Schwartz MK, Kelley JL, Luikart G (2016). Conservation genomics of natural and managed populations: building a conceptual and practical framework. Mol Ecol..

[60] Nielsen R, Paul JS, Albrechtsen A, Song YS (2011). Genotype and SNP calling from next-generation sequencing data. Nat Rev Genet..

[61] Wilson DP (1971). *Sabellaria* colonies at duckpool, North Cornwall, 1961–1970. J Mar Biol Assoc U K..

[62] Firth LB, Mieszkowska N, Grant LM, Bush LE, Davies AJ, Frost MT, Moschella PS, Burrows MT, Cunningham PN, Dye SR, Hawkins SJ (2015). Historical comparisons reveal multiple drivers of decadal change of an ecosystem engineer at the range edge. Ecol Evol..

[63] Curd A, Pernet F, Corporeau C, Delisle L, Firth LB, Nunes FLD, Dubois SF (2019). Connecting organic to mineral: how the physiological state of an ecosystem-engineer is linked to its habitat structure. Ecol Indic..

[64] Buonomo R, Assis J, Fernandes F, Engelen AH, Airoldi L, Serrão EA (2017). Habitat continuity and stepping-stone oceanographic distances explain population genetic connectivity of the brown alga Cystoseira amentacea. Mol Ecol..

[65] Nunes FLD, Van Wormhoudt A, Faroni-Perez L, Fournier J (2017). Phylogeography of the reef-building polychaetes of the genus Phragmatopoma in the western Atlantic region. J Biogeogr..

[66] Mergeay J, Santamaría L (2012). Evolution and biodiversity: the evolutionary basis of biodiversity and its potential for adaptation to global change. Evol Appl..

[67] Maggs CA, Castilho R, Foltz D, Henzler C, Jolly MT, Kelly J, Olsen J, Perez K, Stam W, Vainola R, Viard F, Wares J (2008). Evaluating signatures of glacial refugia for North Atlantic marine organisms. Ecology..

[68] Rowe G, Harris DJ, Beebee TJC (2006). Lusitania revisited: a phylogeographic analysis of the natterjack toad *Bufo calamita* across its entire biogeographical range. Mol Phylogenet Evol..

[69] Teacher AGF, Garner TWJ, Nichols RA (2009). European phylogeography of the common frog (*Rana temporaria*): routes of postglacial colonization into the British Isles, and evidence for an Irish glacial refugium. Heredity..

[70] Jolly MT, Viard F, Gentil F, Thiébaut E, Jollivet D (2006). Comparative phylogeography of two coastal polychaete tubeworms in the Northeast Atlantic supports shared history and vicariant events. Mol Ecol..

[71] Hoarau G, Coyer JA, Veldsink JH, Stam WT, Olsen JL (2007). Glacial refugia and recolonization pathways in the brown seaweed *Fucus serratus*. Mol Ecol..

[72] Provan J, Bennett KD (2008). Phylogeographic insights into cryptic glacial refugia. Trends Ecol Evol..

[73] Scheltema R (1971). Larval dispersal as a means of genetic exchange between geographically separated populations of shallow-water benthic marine gastropods. Biol Bull..

[74] Kettle AJ, Haines K (2006). How does the European eel (*Anguilla anguilla*) retain its population structure during its larval migration across the North Atlantic Ocean?. Can J Fish Aquat Sci..

[75] Cowen RK, Sponaugle S (2009). Larval dispersal and marine population connectivity. Annu Rev Mar Sci..

[76] Jorde PE, Søvik G, Westgaard JI, Albretsen J, André C, Hvingel C, Johansen T, Sandvik AD, Kingsley M, Jørstad KE (2015). Genetically distinct populations of northern shrimp, *Pandalus borealis*, in the North Atlantic: adaptation to different temperatures as an isolation factor. Mol Ecol..

[77] Kool JT, Paris CB, Andréfouët S, Cowen RK (2010). Complex migration and the development of genetic structure in subdivided populations: an example from Caribbean coral reef ecosystems. Ecography..

[78] Foster NL, Paris CB, Kool JT, Baums IB, Stevens JR, Sanchez JA, Bastidas C, Agudelo C, Bush P, Day O, Ferrari R, Gonzalez P, Gore S, Guppy R, McCartney MA, McCoy C, Mendes J, Srinivasan A, Steiner S, Vermeij MJA, Weil E, Mumby PJ (2012). Connectivity of Caribbean coral populations: complementary insights from empirical and modelled gene flow. Mol Ecol..

[79] Kimura M, Weiss GH (1964). The stepping stone model of population structure and the decrease of genetic correlation with distance. Genetics..

[80] Defaveri J, Jonsson PR, Merilä J (2013). Heterogeneous genomic differentiation in marine threespine sticklebacks: adaptation along an environmental gradient. Evolution..

[81] Vera M, Díez-Del-Molino D, García-Marín JL (2016). Genomic survey provides insights into the evolutionary changes that occurred during European expansion of the invasive mosquitofish (*Gambusia holbrooki*). Mol Ecol..

[82] Moore JS, Bourret V, Dionne M, Bradbury I, O’Reilly P, Kent M, Chaput G, Bernatchez L (2014). Conservation genomics of anadromous Atlantic salmon across its North American range: outlier loci identify the same patterns of population structure as neutral loci. Mol Ecol..

[83] Charlesworth D (2006). Balancing selection and its effects on sequences in nearby genome regions. PLoS Genet..

[84] Mable BK (2019). Conservation of adaptive potential and functional diversity: integrating old and new approaches. Conserv Genet..

[85] Fukami HH, Budd AFAF, Levitan DRDR, Jara JJ, Kersanach RR, Knowlton NN (2004). Geographic differences in species boundaries among members of the *Montastraea annularis* complex based on molecular and morphological markers. Evolution..

[86] Nunes FLD, Fukami HH, Vollmer SV, Norris RD, Knowlton N (2008). Re-evaluation of the systematics of the endemic corals of Brazil by molecular data. Coral Reefs..

[87] Le Bras Y, Roult A, Monjeaud C, Bahin M, Quénez O, Hériveau C, Bretaudeau A, Sallou O, Collin O. Towards a life sciences virtual research environment. An e-Science initiative in Western France. JOBIM. 2013:1–10.

[88] Bolger AM, Lohse M, Usadel B (2014). Trimmomatic: a flexible trimmer for Illumina sequence data. Bioinformatics..

[89] Catchen J, Hohenlohe PA, Bassham S, Amores A, Cresko WA (2013). Stacks: an analysis tool set for population genomics. Mol Ecol..

[90] Mastretta-Yanes A, Arrigo N, Alvarez N, Jorgensen TH, Piñero D, Emerson BC (2015). Restriction site-associated DNA sequencing, genotyping error estimation and de novo assembly optimization for population genetic inference. Mol Ecol Resour..

[91] Miller AD, van Rooyen A, Rašić G, Ierodiaconou DA, Gorfine HK, Day R, Wong C, Hoffmann AA, Weeks AR (2016). Contrasting patterns of population connectivity between regions in a commercially important mollusc *Haliotis rubra*: integrating population genetics, genomics and marine LiDAR data. Mol Ecol..

[92] Matz MV (2017). Fantastic beasts and how to sequence them: ecological genomics for obscure model organisms. Trends Genet..

[93] Paris JR, Stevens JR, Catchen JM (2017). Lost in parameter space: a road map for stacks. Methods Ecol Evol..

[94] Jeffries DL, Copp GH, Handley LL, Håkan Olsén K, Sayer CD, Hänfling B (2016). Comparing RADseq and microsatellites to infer complex phylogeographic patterns, an empirical perspective in the Crucian carp, *Carassius carassius*. L. Mol Ecol..

[95] Lischer HEL, Excoffier L (2012). PGDSpider: an automated data conversion tool for connecting population genetics and genomics programs. Bioinformatics..

[96] Excoffier L, Lischer HEL (2010). Arlequin suite ver 3.5: a new series of programs to perform population genetics analyses under Linux and windows. Mol Ecol Resour..

[97] Campbell NR, LaPatra SE, Overturf K, Towner R, Narum SR (2014). Association mapping of disease resistance traits in rainbow trout using restriction site associated DNA sequencing. G3.

[98] Narum SR, Hess JE (2011). Comparison of FST outlier tests for SNP loci under selection. Mol Ecol Resour..

[99] Van Wyngaarden M, Snelgrove PVR, DiBacco C, Hamilton LC, Rodríguez‐Ezpeleta N, Jeffery NW, RRE S, Bradbury IR (2017). Identifying patterns of dispersal, connectivity and selection in the sea scallop, *Placopecten magellanicus*, using RADseq-derived SNPs. Evol Appl..

[100] Pante E, Simon-Bouhet B (2013). marmap: A package for importing, plotting and analyzing bathymetric and topographic data in R. PLoS One..

[101] Mantel N, Valand R (1970). A technique of nonparametric multivariate analysis. Biometrics..

[102] Paradis E, Schliep K (2019). Ape 5.0: an environment for modern phylogenetics and evolutionary analyses in R. Bioinformatics..

[103] Saitou N, Nei M (1987). The neighbor-joining method: a new method for reconstructing phylogenetic trees. Mol Biol Evol..

[104] Jombart T (2008). Adegenet: a R package for the multivariate analysis of genetic markers. Bioinformatics..

[105] Evanno G, Regnaut S, Goudet J (2005). Detecting the number of clusters of individuals using the software STRUCTURE: a simulation study. Mol Ecol..

[106] Earl DA (2012). vonHoldt BM. STRUCTURE HARVESTER: a website and program for visualizing STRUCTURE output and implementing the Evanno method. Conserv Genet Resour..

[107] North EW, Schlag Z, Hood RR, Li M, Zhong L, Gross T, Kennedy VS (2008). Vertical swimming behavior influences the dispersal of simulated oyster larvae in a coupled particle-tracking and hydrodynamic model of Chesapeake Bay. Mar Ecol Prog Ser.

[108] Truelove NK, Box SJ, Aiken KA, Blythe-Mallett A, Boman EM, Booker CJ, Byfield TT, Cox CE, Davis MH, Delgado GA, Glazer BA, Griffiths SM, Kitson-Walters K, Kough AS, Pérez Enríquez R, Preziosi RF, Roy ME, Segura-García I, Webber MK, Stoner AW (2017). Isolation by oceanic distance and spatial genetic structure in an overharvested international fishery. Divers Distrib..

[109] Fox AD, Henry LA, Corne DW, Roberts JM (2016). Sensitivity of marine protected area network connectivity to atmospheric variability. R Soc Open Sci..

[110] Chassignet EP, Hurlburt HE, Smedstad OM, Halliwell GR, Hogan PJ, Wallcraft AJ, Baraille R, Bleck R (2007). The HYCOM (HYbrid Coordinate Ocean model) data assimilative system. J Mar Syst..

[111] Ross RE, Nimmo-Smith WAM, Howell KL (2016). Increasing the depth of current understanding: sensitivity testing of deep-sea larval dispersal models for ecologists. PLoS One..

[112] Kough A, Claro R, Lindeman K, Paris C (2016). Decadal analysis of larval connectivity from Cuban snapper (Lutjanidae) spawning aggregations based on biophysical modeling. Mar Ecol Prog Ser..

[113] Müller M, Haak H, Jungclaus JH, Sündermann J, Thomas M (2010). The effect of ocean tides on a climate model simulation. Ocean Model..

[114] Muir AP, Biek R, Thomas R, Mable BK (2014). Local adaptation with high gene flow: temperature parameters drive adaptation to altitude in the common frog (*Rana temporaria*). Mol Ecol..

[115] Seabra R, Wethey DS, Santos AM, Lima FP (2011). Side matters: microhabitat influence on intertidal heat stress over a large geographical scale. J Exp Mar Biol Ecol..

[116] Bernatchez S, Xuereb A, Laporte M, Benestan L, Steeves R, Laflamme M, Bernatchez L, Mallet MA (2019). Seascape genomics of eastern oyster (*Crassostrea virginica*) along the Atlantic coast of Canada. Evol Appl..

[117] Seabra R, Wethey DS, Santos AM, Lima FP (2015). Understanding complex biogeographic responses to climate change. Sci Rep..

[118] Lima FP, Wethey DS (2009). Robolimpets: measuring intertidal body temperatures using biomimetic loggers. Limnol Oceanogr Methods..

[119] Coop G, Witonsky D, Di Rienzo A, Pritchard JK (2010). Using environmental correlations to identify loci underlying local adaptation. Genetics..

[120] Bernatchez S, Laporte M, Perrier C, Sirois P, Bernatchez L (2016). Investigating genomic and phenotypic parallelism between piscivorous and planktivorous lake trout (*Salvelinus namaycush*) ecotypes by means of RADseq and morphometrics analyses. Mol Ecol..

[121] Lotterhos KE, Whitlock MC (2015). The relative power of genome scans to detect local adaptation depends on sampling design and statistical method. Mol Ecol..

[122] Wenzel MA, Piertney SB (2014). Fine-scale population epigenetic structure in relation to gastrointestinal parasite load in red grouse (*Lagopus lagopus scotica*). Mol Ecol..

[123] Schweizer RM, VonHoldt BM, Harrigan R, Knowles JC, Musiani M, Coltman D, Novembre J, Wayne RK (2016). Genetic subdivision and candidate genes under selection in North American grey wolves. Mol Ecol..

[124] De La Torre AR, Roberts DR, Aitken SN (2014). Genome-wide admixture and ecological niche modelling reveal the maintenance of species boundaries despite long history of interspecific gene flow. Mol Ecol..

[125] Muir AP, Dubois SF, Ross RE, Firth LB, Knights AM, Lima FP, Seabra R, Corre E, Le Corguillé G, Nunes FLD. Data from: Seascape genomics reveals population isolation in the reef-building honeycomb worm, *Sabellaria alveolata* (L.). NHBS SRA. 2020; https://www.ncbi.nlm.nih.gov/bioproject/645800. 10.1186/s12862-020-01658-9 PMC7418442 32778052

[126] Muir AP, Dubois SF, Ross RE, Firth LB, Knights AM, Lima FP, Seabra R, Corre E, Le Corguillé G, Nunes FLD. Data from: Seascape genomics reveals population isolation in the reef-building honeycomb worm, *Sabellaria alveolata* (L.). Figshare. 2020; https://figshare.com/articles/dataset/Seascape_genomics_reveals_population_isolation_in_the_reef-building_honeycomb_worm_Sabellaria_alveolata_L_/12645896. 10.1186/s12862-020-01658-9 PMC7418442 32778052

