# Supplementary material for: Seascape genomics reveals population isolation in the reef-building honeycomb worm, Sabellaria alveolata (L.)
Source: BMC Evol Biol. 2020 Aug 10;20:100. doi: 10.1186/s12862-020-01658-9 (PMC7418442; doi:10.1186/s12862-020-01658-9)
Supplement: Supplementary file 1 — Additional file 1. SeascapeGenomics_Muiretal. [file 12862_2020_1658_MOESM1_ESM.docx]

**Additional file 1:**

**Seascape genomics reveals population isolation in the reef-building honeycomb worm, *Sabellaria alveolata***

Anna P. Muir, Stanislas F. Dubois, Rebecca E. Ross, Louise B. Firth, Antony M. Knights, Fernando P. Lima, Rui Seabra, Erwan Corre, Gildas Le Corguille and Flavia L. D. Nunes.

Table 1: Proportions (on a scale of 0-1.0) of modelled larval settlement at sampled sites.

|  |  | SETTLEMENT SITES | | | | | | | | |
| --- | --- | --- | --- | --- | --- | --- | --- | --- | --- | --- |
|  |  | Northern Irish Sea | North Atlantic | Southern Irish Sea | English Channel | Bay of Biscay | Tyrrhenian Sea | Balearic Sea | Iberian Peninsula | North Africa |
| SOURCE POPULATIONS | Northern Irish Sea | 0.21 |  | 0.00066 |  |  |  |  |  |  |
|  | North Atlantic |  | 0.79 |  |  |  |  |  |  |  |
|  | Southern Irish Sea | 0.00014 |  | 0.18 |  |  |  |  |  |  |
|  | English Channel |  |  |  | 0.50 |  |  |  |  |  |
|  | Bay of Biscay |  |  |  |  | 0.23 |  |  |  |  |
|  | Tyrrhenian Sea |  |  |  |  |  | 0.18 |  |  |  |
|  | Balearic Sea |  |  |  |  |  |  | 0.17 |  |  |
|  | Iberian Peninsula |  |  |  |  | <0.0001 |  |  | 0.07 |  |
|  | North Africa |  |  |  |  |  |  |  | <0.0001 | 0.07 |

Shown is the most optimistic scenario (daily releases throughout the years of simulation, 12 weeks PLD). Note this table differs from F_ST_ pairwise plots as it shows directional flow of a single generation of dispersal (rather than pairwise genetic dissimilarity which is non-directional): row names represent source populations, and columns settlement sites. Very limited to no connectivity is observed between all sites.

Figure 1: Plot of reads per site, created in R version 3.1.2 [99].


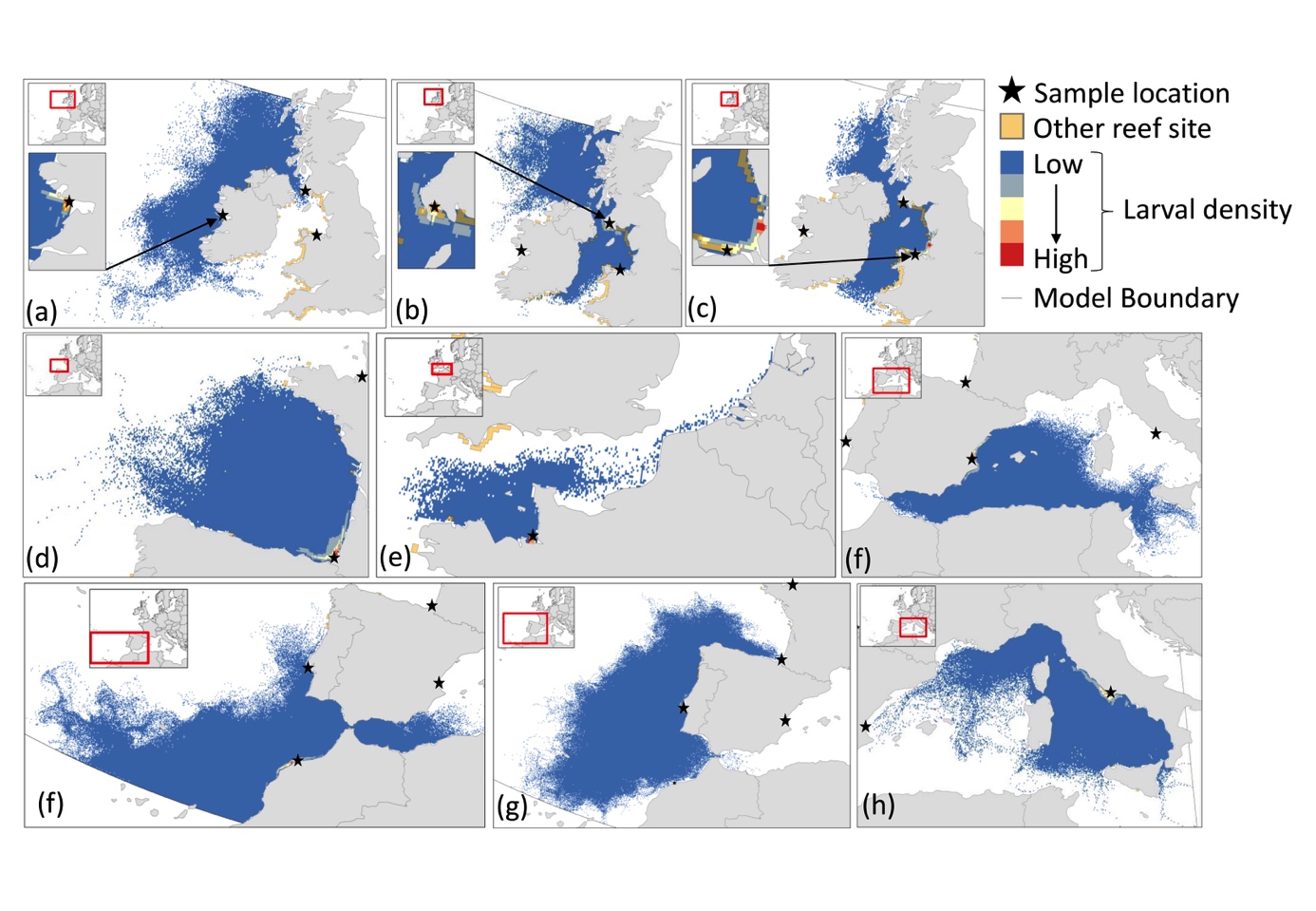


Figure 2: Per site maps of larval density to visualise the pathways of simulated dispersal in the most optimistic scenario. Shown are the daily releases throughout the year, 12 weeks PLD. Genetic sampling locations are shown as stars, with releases from (a) North Atlantic, (b) Northern Irish Sea, (c) Southern Irish Sea, (d) Bay of Biscay, (e) English Channel, (f) Balearic Sea, (g) North Africa, (h) Iberian Peninsula, (i) Tyrrhenian Sea. Other known reef sites, sourced from GBIF, are indicated by 10 km boxes. Created in Matlab (Mathworks, Version R2016a).
